# Application of CRISPR/Cas9 for biomedical discoveries

**DOI:** 10.1186/s13578-015-0027-9

**Published:** 2015-06-21

**Authors:** Sean M. Riordan, Daniel P. Heruth, Li Q. Zhang, Shui Qing Ye

**Affiliations:** Department of Pediatrics, The Children’s Mercy Hospital, Kansas City, MO USA; Department of Biomedical and Health Informatics, University of Missouri Kansas City School of Medicine, Kansas City, MO USA

**Keywords:** CRISPR, Cas9, Genome editing, Animal model

## Abstract

The Clustered Regions of Interspersed Palindromic Repeats-Cas9 (CRISPR/Cas9), a viral defense system found in bacteria and archaea, has emerged as a tour de force genome editing tool. The CRISPR/Cas9 system is much easier to customize and optimize because the site selection for DNA cleavage is guided by a short sequence of RNA rather than an engineered protein as in the systems of zinc finger nucleases (ZFN), transcription activator–like effector nucleases (TALEN), and meganucleases. Although it still suffers from some off-target effects, the CRISPR/Cas9 system has been broadly and successfully applied for biomedical discoveries in a number of areas. In this review, we present a brief history and development of the CRISPR system and focus on the application of this genome editing technology for biomedical discoveries. We then present concise concluding remarks and future directions for this fast moving field.

## Introduction

Targeted genome engineering provides the ability to precisely modify genetic information in order to study gene function, biological mechanisms, and disease pathology. Historically, random mutagenesis or low-efficiency homologous recombinations were used to modify the genomes of cell lines or animal models. However, new advances in the design of sequence-specific endonucleases have enabled more effective, targeted editing of the genome.

The first prevalent method for targeted genome editing utilizes zinc finger nucleases (ZFN) [1–3]. ZFN are proteins capable of binding to specific regions of DNA. These nucleases consist of a zinc finger protein bound to the cleavage domain of the restriction enzyme *Fok*I [4]. Meganucleases, though not widely adapted, are restriction endonucleases that bind larger than normal (12 bp) sequences of DNA [5, 6]. Transcription activator–like effector nucleases (TALENs) are engineered proteins with a sequence-specific DNA-binding domain fused to a nonspecific DNA-cleaving nuclease [7, 8]. Finally, the most recent and fastest growing method for genome editing is based on the Clustered Regions of Interspersed Palindromic Repeats (CRISPR) viral defense system found in bacteria and archaea [9].

The common factor in all of these methods is the necessity to introduce a double stranded break (DSB) in genomic DNA. When DSBs occur naturally, cells respond by activating DNA repair machinery. Utilization of these repair processes allows for either homology directed repair (HDR) or non-homologous end joining (NHEJ) recombination resulting in the insertion, deletion, or mutation of specific DNA sequences [10]. Thus, the ability to generate DSBs at targeted genomic locations, coupled with DNA repair mechanisms, has revolutionized genome editing.

The development of ZFN, TALEN, and to some extent meganuclease, technologies requires the design of custom endonucleases to target specific regions of DNA. Although these methods have been used to edit genomes successfully, there remain significant technical drawbacks. First, the development of custom proteins for the recognition of specific DNA sequences is cumbersome and expensive. Second, the optimization of these custom proteins is very time consuming. In contrast, the CRISPR system is easy to customize and optimize because the site selection for DNA cleavage is guided by a short sequence of RNA rather than an engineered protein. The short CRISPR RNA molecule (crRNA), also called a guide RNA (gRNA), utilizes standard Watson-Crick binding to recognize the target sequence, termed the protospacer, and position a CRISPR-associated nuclease (Cas) to create the DSB. This feature not only makes it easy to develop targeted modifications of a single gene, but it also facilitates the development of functional genetic screens using libraries of gRNAs targeting thousands of genes [11, 12]. In this review, we present a brief history and development of the CRISPR system and focus on the application of this genome editing technology for biomedical discoveries followed by concluding remarks with a look toward possible future directions for this popular technology.

### History of CRISPR

Ishino et al. initially discovered the CRISPR architecture in the 1980s when they noticed an “unusual structure” in the 3′ flanking region of the *Escherichia coli iap* gene [13]. The region contained 5 highly homologous 29 base pair (bp) nucleotide sequences separated by 32 bp nucleotide variable regions. Over the next decade additional examples of CRISPR loci were identified as more and more bacterial genomes were sequenced. The CRISPR acronym itself was proposed in 2002 by Jansen and Mojica [14]. It wasn’t until 20 years after their initial discovery that the spacer sequences located within the CRISPR repeats were shown to confer resistance to specific bacteriophage introduced to the bacterial strain *Streptococcus thermophilus,* a commonly used bacteria in the dairy industry [9]. These initial observations have since been confirmed in other organisms and now, the unique spacer regions in the CRISPR loci are understood to be a type of immune memory system to protect against invading phage or plasmid DNA. Through this system the bacteria are able to extract a short sequence from the invading DNA and file it away in the CRISPR locus where it can be accessed later by transcription. Recent work utilizing CRISPR/Cas loci deficient *Staphylococcus aureus* transformed with the commonly used *Streptococus pyogenes* CRISPR locus has shown that multiple *cas* genes are important for the initial identification and excision of invading DNA [15].

While the role of the Cas proteins is now better understood, their role in CRISPR based resistance was originally a mystery. Structural analysis of different Cas proteins identified homology to known endonuclease domains, suggesting a possible role in conferring viral resistance through the introduction of DSBs. During activation of the CRISPR response the unique spacer sequences are transcribed into short crRNAs. Garneau et al., was one of the first groups to show crRNA worked with Cas proteins to lead to DSBs in invading DNA [16]. The identification and characterization of different *cas* genes in multiple bacteria and archaea lead to the classification of three major CRISPR types (I, II and III). The categorization of the *cas* genes was first proposed by Haft et al. [17] and later updated by Makarova et al. [18]. Evidence that Cas9 was an RNA guided endonuclease with independent nuclease domains responsible for cutting both strands of DNA was presented later by Jinek et al. [19]. This was followed by the direct confirmation of this interaction through the solving of the crystal structure of the Cas9- guideRNA-target DNA complex [20]. Since this discovery numerous labs have modified the system to improve it by reducing off-site targeting [21] or utilizing it for alternative goals such as affecting gene transcription [22] or creating large libraries for functional screens [11, 12].

**Fig. 1:**
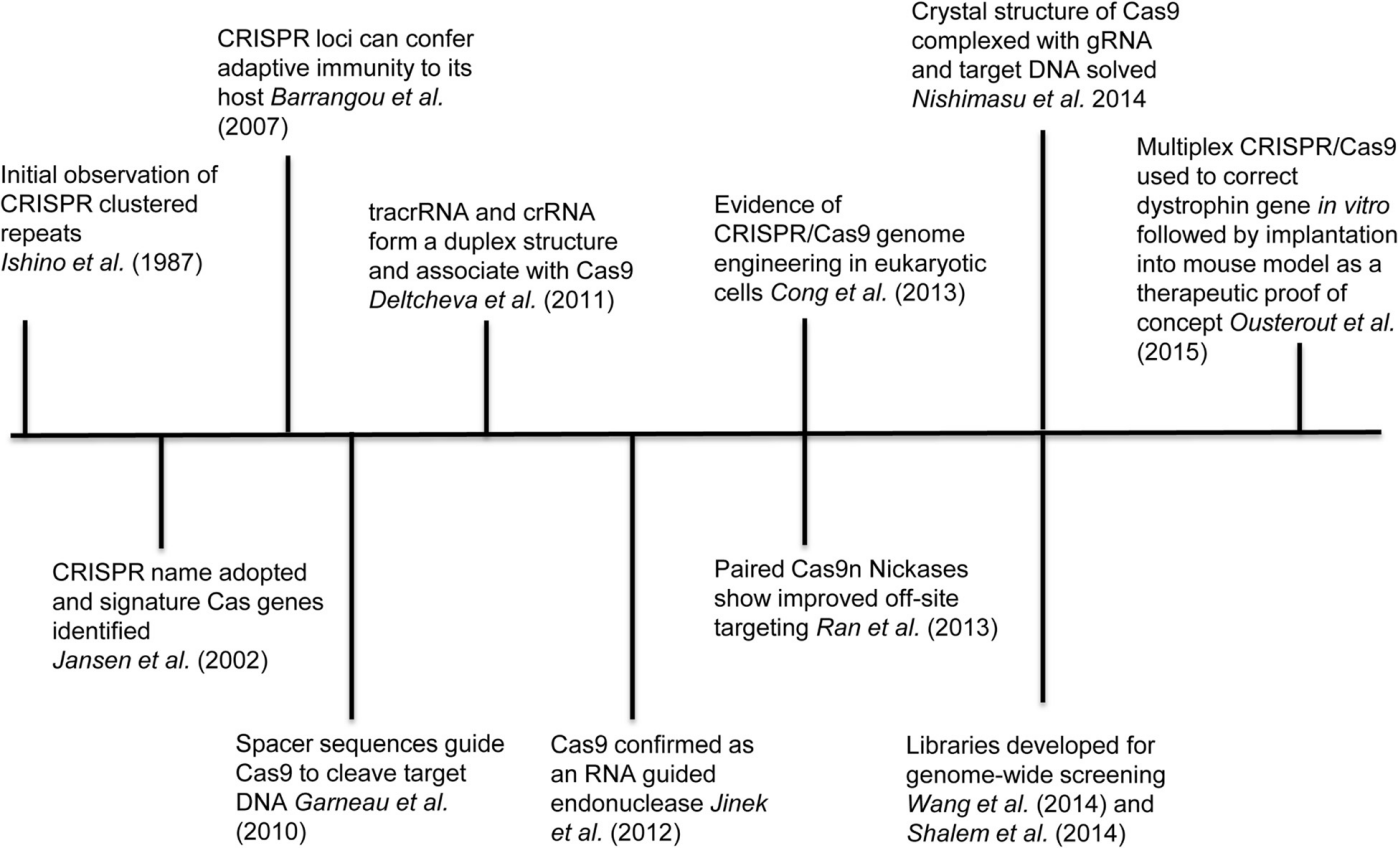
History of CRISPR/Cas development. A useful understanding of the potential and capabilities of the CRISPR/Cas system took many years to develop. Only recently has there been an explosion in the development and application of this system

Although there is great diversity in the CRISPR/Cas systems, they all share three common stages: adaptation, crRNA biogenesis and immunity. Adaptation is the process of acquiring unique spacer sequences from viral and plasmid DNA. crRNA biogenesis involves the transcription and processing of crRNA. Immunity, also termed interference, entails the formation of an endonuclease complex capable of recognizing and digesting invading phage and plasmid DNA. The discovery of endonucleases that could be guided to specific regions of the genome was met with immediate interest. Successful attempts to develop easy to use genome editing methods lead to the creation of chimeric RNA systems containing crRNA and the trans activating RNA (tracrRNA) to create a single guide RNA (sgRNA) to be used with the Cas protein, Cas9 [23, 24]. Since then researchers at the Broad Institute of MIT and Harvard have been awarded a U.S. Patent for the technology, which they have licensed to multiple biotechnology companies leading to a huge growth in the technology and its availability. A summary of the history of CRISPR/Cas technological development can be seen in Fig. 1.

### CRISPR Types I and III

As an increasing number of bacteria and archaea were found to contain CRISPR loci, different mechanisms of immunity, often times species specific, immerged. The CRISPR locus is composed of unique pre-crRNA sequences obtained from invading DNA inserted between a series of direct repeats (~20-50 bp) [25, 26]. The pre-crRNA sequence is transcribed, processed into mature crRNAs, and assembled into an interference complex with one or more Cas proteins. The *cas* genes, which are organized as an operon, are located upstream of the CRISPR locus promoter and are important for both information processing and the subsequent interference. The three main CRISPR types consist of 10 subtypes; 5 type I, 3 type II and 2 type III [27, 28]. The CRISPR/Cas systems differ in the organization of *cas* genes into operons, as well as the nature of the repeats in the CRISPR array. These structural features dictate how the pre-crRNA is processed, how the components of the interference complex are assembled, whether there is secondary processing of the complex, and how the interference complex recognizes and cuts its target (Table 1). The type II system is the best understood of the three CRISPR systems, due largely to the fact that only one Cas protein, Cas9, is required for introducing DSBs [9]. The relative simplicity of the type II system has lead to its adoption into a ready-made tool for genome manipulation. Because this review focuses on the current state of application of the CRISPR/Cas9 system in basic and translational research, we will not present detailed information about types I and III. Those interested in these systems should read the recent excellent review from Hochstrasser and Doudna [27].

**Table 1:** Major components of each CRISPR type^a^

Action	Type I	Type II	Type III
Spacer acquisition	Cas1/Cas2	Cas1/Cas2/Csn1/Cas9	Cas1/Cas2
Pre-cRNAprocessing	Cas6 or Cas5	Rnase III	Cas6
Interference Complex members	Cascade	tracrRNA/Cas9	Csm/Cmr
Secondary processing	None	5′ end cleavage	3′ end cleavage
Interference Subtubes	I-A, I-B, I-E, I-F, I-C,	II-A, II-B, II-C	III-A, III-B

^a^CRISPR types are characterized by their operon composition the involved Cas proteins and the structure of the repeats in the CRISPR array. This table presents the defining characteristics of each type [27, 28]. Cascade = CRISPR-associated complex for antiviral defense

### CRISPR – Type II

**Fig. 2:**
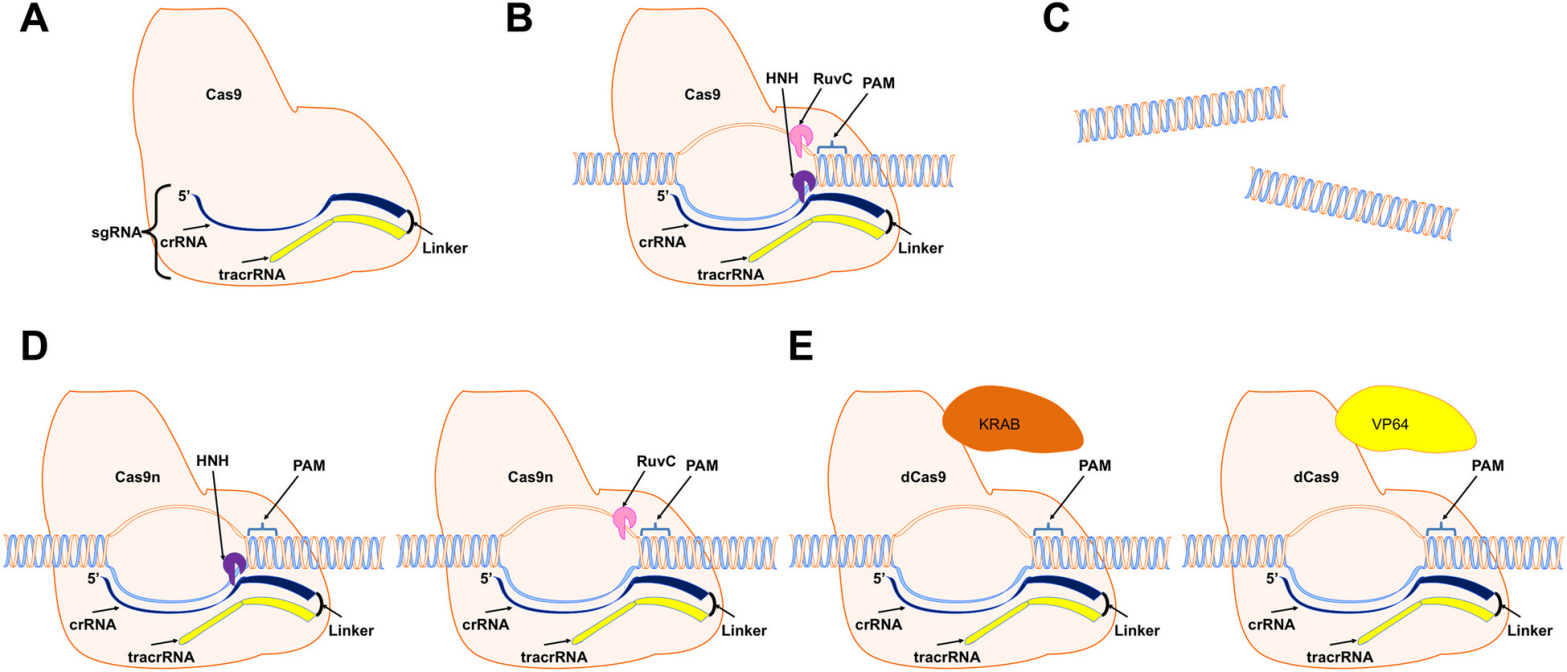
Mechanism of Cas9 action. **a** The three essential components necessary for CRISPR/Cas activity, using spCas9 as an example, are Cas9, crRNA and tracrRNA. The introduction of the linker region to combine the crRNA and tracrRNA into a single guide RNA (sgRNA) improves overall targeting efficiency. **b** Recognition of the protospacer region by the sgRNA in conjunction with Cas9 recognition of the appropriate PAM sequence initiates cleavage. **c** Cleaved DNA fragments. **d** Mutated forms of Cas9 can contain a single active domain to act as a Cas9n “nickase” where only one strand of the DNA is cleaved. **e** Alternatively both catalytic domains can be mutated to form a dCas9 “dead” nuclease that can then be tethered to effector molecules to target repression or activation, KRAB and VP64 respectively, for example. Images adapted from motifolio.com

The CRISPR system loci is defined by the presence of the 20 – 50 base pair repeats separated by the unique spacer sequences acquired from invading viral and plasmid DNA [29]. In the type II CRISPR system, spacer acquisition depends on Cas1, Cas2, Csn2, and Cas9. While Csn2 and Cas9 are unique to the type II system, Cas1 and Cas2 seem to be common to all three CRISRP types [15, 18]. For the type II system there is evidence that Cas9 is integral in the initial recognition of the potential protospacer + PAM sequence in the invading DNA [15]. This dual role for Cas9 is a logical mechanism to ensure that the protospacers selected will be present next to the required PAM sequence so that Cas9 will be able to recognize it for future targeting and cleavage when challenged. Following transcription, RNAse III and Cas9 process the pre-crRNA transcript into the mature crRNA forms [30]. In addition to processing the pre-crRNA transcript, Cas9 also functions as the sole endonuclease necessary for the introduction of DSBs in the Type II CRISPR system. Cas9 is guided to its target sequence after forming a complex with the crRNA and a tracrRNA. TracrRNA forms a duplex with the crRNA and is essential for the proper Cas9:crRNA interaction and recognition of the target DNA sequence (Fig. 2) [30].

Cas9 cleavage depends upon the presence of a protospacer adjacent motif (PAM) in the target DNA. PAM recognition sequences can vary greatly between bacteria. For genome engineering purposes, the most commonly used Cas9, SpCas9, comes from *Streptococcus pyogenes* [23]. SpCas9’s popularity is largely based on its short PAM recognition sequence, 5′-NGG-3′ or at a lower frequency, 5′-NAG-3′ [31] allowing for increased probability of having a potential protospacer located at the desired location of the gDNA. However, additional Cas9’s have been isolated from other bacteria and used for CRISPR genome engineering. Examples of additional tested Cas9s include NmCas9 and SaCas9 from *Neisseria menigitidis* and *Staphylococcus aureus,* respectively. NmCas9’s PAM sequences are 5′-NNNNGATT-3′ or 5′-NNNNGCTT-3′. SaCas9’s PAM most efficient sequence is 5′-NNGRRT-3′ though all 5′-NNGRR-3′ can be cleaved (R = A or G) [32, 33]. The smaller size of SaCas9 is better suited for viral delivery than spCas9 and therefore, is especially advantageous for *in vivo* work [33]. The PAM site must be located immediately 3′ of the protospacer in the DNA for proper recognition by the PAM binding domain in Cas9 [19, 34, 35]. Cas9 contains two cleavage domains, HNH and RuvC, and each is responsible for cleaving one strand of the DNA when bound (Fig. 2b) [19]. The PAM sequence is necessary for both initial protospacer acquisition and for later interference. The PAM requirement increases the specificity of the guidance system and blocks the ability of the complex to cut the CRISPR loci.

### Development of CRISPR Cas9 for molecular biology

Once the necessary components for RNA guided endonuculease activity were determined, the race was on for the development of these components into a customizable system for the introduction of targeted DSBs into the genomes of various organisms. The use of the Cas9 system for genetic alteration centers on the repair mechanism that is initiated by the introduction of the DSB by Cas9. When DSBs occur in the genome the breaks can be repaired along two possible pathways, NHEJ and HDR. The first pathway, NHEJ, involves the quick ligation of the blunt ends of DNA remaining after DSBs occur. There are two subtypes of NHEJ, canonical and alternative end joining [36]. The canonical pathway results in relatively few errors or small deletions. However, if this pathway fails, the more error prone Ligase I or Ligase III-dependent end joining will take over and will likely lead to one or more of a variety of mutations. These random mutations can cause insertions or deletions (indels) that could alter open reading frames and insert premature stop signals resulting in a gene knockout.

The second pathway, HDR, normally uses a sister chromatid as a homologous template to repair DNA damage. In eukaryotic cells the occurrence of HDR is extremely low, in part due to the much more prevalent NHEJ. For genome targeting, the HDR repair mechanism can be subverted by introducing custom template DNA containing homologous arms on either side of a DSB site. This technique can be used to insert novel DNA or remove portions of the genome allowing for a multitude of custom changes, including gene insertion, gene deletion, gene mutation, promoter activation or suppression, SNP alteration, and others [37, 38]. To increase the efficiency of HDR for use with CRISPR, researchers have suppressed a number of key factors in the NHEJ pathway, which has lead to 4 – 8 fold higher efficiency of HDR in mammalian cells [39]. For those looking for more information on HDR, an excellent review by Heyer et al. can be found here [40].

The specific nature of CRISPR/Cas9 allows for multiple genes to be targeted and repaired simultaneously. CRISPR multiplexing was first performed *in vitro* with individual plasmids, but can now be accomplished more conveniently with a single plasmid containing multiple sgRNAs [41]. Additional groups are working on harnessing the CRISPR array system in *E. coli* for easier multiplexing [42]. Recently, Ma et al. simultaneously mutated four different genes *in vivo* [43]. Additional systems shown to be capable of CRISPR multiplexing include: bacteria [44], plants [45], yeast, [46] pigs [47], frogs [48] and zebra fish [49, 50]. For diseases where there can be one or more mutations, such as Duchenne muscular dystrophy (DMD), the ability to introduce multiple sgRNAs simultaneously has proven to be an effective strategy [51].

### Shortcomings of CRISPR Cas9

While the ability to use a gRNA to actively target a region of the genome for the introduction of a DSB is a huge accomplishment, this technology is not without its shortcomings. The first of these shortcomings is the requirement of a PAM sequence for the proper recognition of a target site by the gRNA. While this requirement does increase the specificity of the system, it has also decreased the flexibility in the design of gRNAs for the purpose of genome editing. However, the PAM site varies in both length and complexity, depending on the CRISPR type and species, allowing for increased flexibility in gRNA design [52]. In addition, if the donor sequence to be used for HDR contains both the full target site and the PAM site, then the efficiency of HDR could suffer as the donor sequence would also be targeted along with the genomic DNA [53]. The most common method to avoid this problem is to introduce silent point mutations in the PAM site within the donor sequence so that it will no longer be recognized. However, this solution does not allow for situations where point mutations cannot be tolerated, such as when point mutations themselves are being studied for their effects on a promoter’s transcription enhancement capabilities. In this instance additional point mutations could cause unforeseen effects on the binding of transcription factors to the DNA.

The second shortcoming of the CRISPR Cas9 system is the presence of unintended or “off-target” effects of the gRNA. Because DSBs can lead to the introduction of indels through the process of NHEJ, off-target effects have the potential to introduce secondary and potentially harmful mutations, which could possibly cause either a reduction or increase in the production of a crucial gene. Strategies, such as improving algorithms for gRNA design and using paired Cas9 nickases (Cas9n), where one of the catalytic domains are inactivated to increase DSB specificity, have proven effective in reducing off-target effects to very low or even undetectable levels [23].

An alternative strategy to using nickases to reduce off-target effects is through the use of a “dead” Cas9 (dCas9) where both catalytic domains are inactivated [22]. While dCas9 no longer has the ability to cleave, it can still complex with the gRNA and bind to DNA, and as such, it has proved to be an extremely useful molecular tool for targeting other types of proteins to a specific region of DNA. This modification has provided an additional method for reducing off-target affects through the fusion of the *FokI* catalytic domain to dCas9 (fCas9) [54, 55]. FokI is the same catalytic domain used in the ZFN and TALEN systems of genome engineering. Similar to Cas9n, fCas9 requires two separate guide RNAs to simultaneously bind to one strand of DNA create a DSB and showed a similar increase in specificity with Cas9n over wild type Cas9. In addition, the use of the *FokI* domain should reduce unintended modifications because of the more rigid spatial requirements for cutting and the lack of activity as a monomer that can occur with Cas9n.

### Applications of the CRISPR/Cas9 system

The CRISPR/Cas9 system has emerged as a tour de force genome editing tool. It has been broadly and successfully applied for biomedical discoveries in a number of areas, a summary of which can be seen in Table 2. The following section is not intended to comprehensively catalog the full spectrum of its applications but highlight several major examples of such rendering.

**Table 2:** Summary of CRISPR/Cas9 applications

The original application of the CRISPR/Cas, **Genome Engineering**, has proven to be extremely useful in the investigation of numerous genetic features and the improved creation of transgenic animals. However, the modification of the Cas protein has lead to the development of additional applications including: gene **Activation** and **Repression** through the use of effector molecules tethered to inactive Cas9, **Functional Screening** through libraries of guide RNAs to introduce NHEJ based InDels or through activation/repression dCas9, **Inducible Regulation** through the use light or chemical induction for more specific control, **DNA labeling** through fluorescently labeled dCas9, **Isolation** of specific genomic regions through epitope labeled dCas9 and finally the ability to introduce multiple sgRNAs and or various Cas9 proteins with different PAM site requirements allows for more complicated **Multiplexing** experimental designs. The power of these various applications is enhanced by their utility both *in vitro* and *in vivo* as these techniques have been shown to be effective in a wide variety of cell lines and animal models	

### CRISPR/Cas9 regulation (as an alternative to RNAi)

One interesting repurposing of the CRISPR/Cas9 technology has been through modulation of the transcription of various genetic targets. Current methods for transcriptional repression and activation such as RNAi and other DNA binding proteins, ZFN and TALEN, harbored significant shortcomings such as off-target effects, toxicity, and in the case of ZFN and TALEN proteins, difficulty with design and implementation. One of the first attempts at CRISPR/Cas9 based interference was with dCas9. dCas9 is useful because, though it is catalytically inactive it can still successfully target DNA. When researchers targeted the promoter region of a gene of interest it could cause between 100 and 300 fold repression in *E. coli* [22, 56], presumably through blocking the binding of RNA polymerase due to steric hindrance. This CRISPR based interference (CRISPRi) not only showed high levels of repression but also no evidence of off-target effects by RNA-seq. Interestingly, multiple gRNAs targeting the same gene that were simultaneously introduced had a combinatorial effect on repression and could increase the level of knockdown to nearly 1000-fold [56]. In the mammalian HEK cell line the effect was not as potent as these cells showed at most about a 2-fold expression difference and had a significant number of sgRNAs that showed no effect at all [56]. In addition to repression these first studies also explored the role that dCas9 could play in activation (CRISPRa) by introducing a dCas9 fused to the RNA polymerase ω subunit capable of causing up to 23-fold increase in expression [22]. Alternatively, a tetramer of the herpes simplex activation domain VP16, termed VP64, has been shown to be effective activation inducing effector molecule when tethered to dCas9 [57]. The Zhang lab has optimized the use of VP64 for activation through modification of both the sgRNA design and addition of additional helper molecules, MS2, P65 and HSF1, to create a new system called the synergistic activation mediator (SAM) [58].

Follow up studies on CRISPRi showed that the fusion of repressor effector proteins such as the KRAB (Kruppel-associated box) domain of Kox1 to dCas9 could increase the effectiveness of repression of reporter gene activity [59]. CRISPRi was also shown to be effective at repressing microRNA in murine cells [60]. Further study of the ability of effector proteins bound to dCas9 to alter genes related to ricin resistance has lead to a better understanding of the rules governing both CRISPRa and CRISPRi repression, leading to the development of a CRISPRi/CRISPRa library screening system [61]. This system has several advantages over other indel inducing CRISPR based loss-of-function library systems. As an inducible and reversible, genome scale-screening platform without detectable intrinsic toxicity the CRISPRi/a system should prove to be an important tool for many areas of research moving forward [61].

### CRISPR/Cas9 for improved creation of animal models

The characterization of disease phenotypes is a major goal of mouse geneticists. Analyzing genetic mutations in a whole animal may support and validate results found in cell culture. Since the first transgenic mice were made in 1981, the process has been improved, resulting in a more versatile and user-friendly method. The introduction of Bacterial Artificial Chromosomes (BAC) in the 1990s allowed for the use of larger constructs. Traditional methods to create mouse models with an altered genetic makeup can take from 8 to 13 months of work, which is a considerable investment of both time and resources. By comparison, CRISPR/Cas9 was used to create transgenic mice with an observable albino phenotype after a single microinjection of C57BL/6 J mouse embryos [62].

Seminal work by the Huang group showed that genetic engineering in eukaryotes was possible by microinjection of CRISPR/Cas9 mRNAs into zebrafish embryos. They then took this a step further by showing that this technique could be used to delete a portion of EGFP in an established transgenic mouse [63]. Since this initial proof of concept experiment, numerous groups have continued to advance the field of transgenic mouse models created by CRISPR/Cas9. Wang et al. showed that multiplexed targeting of numerous genes simultaneously could create a complex mouse model in with a single injection [64]. Wu et al. was one of the first groups to show that it was possible to correct a genetic disease *in vivo* when they rescued a cataract phenotype by correcting a mutated *Crygc* gene [65]. In addition, CRISPR/Cas9 can be used to delete up to 10 kb of the genome via only 2 gRNAs [66]. Mutated Cas9n nickases have also been shown to effectively facilitate the creation of both knock-in and knock out mouse models with the added benefit of reduced levels of off-target effects [67].

Another problem that researchers often have to deal with is the production of recessive homozygous loss-of-function mutations. To that end Gantz and Bier developed an autocatalytic mutation system they call mutagenic chain reaction (MCR) [68]. MCR consists of a vector where Cas9 and sgRNA are flanked by two homology arms targeting the region to be cut. This system was capable of effectively distributing itself in both somatic and germline cells in *Drosophila* for the creation of homologous mutant flies that would normally have not been possible by mendellian inheritance. MCR could prove to be extremely useful though the authors do note that because of its ability to act automatically there is a biological risk associated if these animals were accidentally released into the environment. They recommend strict barrier containment protocols and increased dialogue within the scientific community about the safety measures necessary for these types of tools [68].

### Inducible CRISPR control

While editing or modifying the expression of particular regions of an organism’s genome in every tissue and cell may be a strategy for some basic and therapeutic applications of RNA guided genome editing, there are situations where these changes would need to be controlled more directly. For example, doxycycline inducible CRISPR (iCRISPR) has been developed to allow for genome targeting at later stages of mouse development. This technology allows for the limitation of the duration of Cas9 expression in a biallellic mutation in multiple target loci [69]. Work in Feng Zhang’s lab at the Broad Institute established that light controlled, reversible, genome activation was possible through the use of a two hybrid TALEN system they termed light inducible transcriptional effectors (LITES) [70]. Though this optogenetic system is effective it does not allow the same flexibility in targeting as the newly developed light-activated CRISPR-Cas9 effector system (LACE) [71]. Both of these systems use blue light and the activator proteins cytochrome 2p with its interacting partner CIB1 to accomplish activation with LACE utilizing the inactive dCas9 protein. The ease with which the LACE system can be redesigned allows for use in difficult systems, such as those where multiple effector regions in a promoter need to be targeted. Through the development of this system researchers found they could increase the overall activation of a gene by including multiple gRNAs targeting the promoter and by fusing the N terminal fragment of CIB1 (CIBN) to both the 5′ and 3′ end of dCas9. Fusing to both ends of dCas9 resulted in a 10 – 100 fold increase in activation compared to the single fusion version [71].

### CRISPR/Cas9 for SNP analysis *in vivo*

The first group to analyze individual point mutations with CRISPR/Cas9 *in vivo* was the Takada group in Tokyo, Japan [72]. The time and cost limitations of creating a mouse before CRISPR initially prevented the *in vivo* investigation of SNPs. This limitation means that the majority of the work done in this field has not been sufficiently validated. The Takada group was able to show that by microinjecting synthesized RNAs and single-strand oligodeoxynucleotide (ssODN) as a donor template for HDR into mouse zygotes they could introduce defined point mutations in the mouse genome [72]. This technique could be used to study the role of SNPs in other non-coding regions of the genome, such as a transcription factor binding sites in a promoter or a distal enhancer region. However, the wild type Cas9 showed some off-target effects, so the researchers used the hCas9 D10A nickase along with variously placed ssODNs. Interestingly, they found that even though they were able to successfully introduce the desired point mutation, HDR was only able to incorporate the portion of the donor sequence that was in close proximity to the DSB site [72].

### Additional animal models being developed

In addition to the large number of mouse models being created with CRISPR/Cas9 technology, additional organisms have proven responsive to this method of manipulation, including traditional animal models: *Drosophila melanogaster* [73, 74], *Caenorhabditis elegans* [75, 76], *Saccharomyces cerevisiae* [77] and *Dani rerio* [78, 79]. CRISPR/Cas9 genetic engineering has also proven to be possible in rats whose limited genetic toolbox was a large reason to avoid this commonly used model organism [80, 81]. In rats, multi-gene targeting and conditional allele modifications have been performed successfully in the generation of new transgenic models [82, 43].

Importantly, CRISPR/Cas9 technology is also proven to be effective in non-traditional animal models, like goats and pigs, which are important for both agricultural and biomedical research and development [83–88]. This technology has even allowed for simplified genetic modification [89] and creation of disease models [90] in non-human primates. These results indicate the potential of CRISPR/Cas9 to greatly improve our ability to model diseases in animals more closely related to humans. Another interesting example of how this technology is opening new avenues of research can be seen in the development of a new animal model, the short-lived African turquoise killifish *Nothobranchius furzeri*. The killifish is an attractive alternative to other vertebrate models used for age-related diseases such as rodents or zebrafish. The shorter lifespan (4 – 6 months) allows for an overall reduction in the time necessary for the manifestation of an overt aging phenotype that includes common aging biomarkers [91]. In addition, the killifish has practical advantages such as lower maintenance costs and the rapid production of offspring. When these advantages are coupled with the ability to manipulate the genome through CRISPR/Cas9 the potential for discovery in age-related diseases is significantly enhanced [92].

### Screening studies with CRISPR

Pioneering works from multiple groups lead to the rapid production of pooled sgRNA libraries with the ability to alter gene production for the purpose of screening cell lines through either a positive or negative toxicity screen. These techniques either induce random indels that will likely lead to the disruption of a gene product, available in both humans [11, 12, 93] and mice [94], or through inactive dCas9 coupled to either repressors or activators [58, 61]. Chen et al. used a CRISPR screen to identify novel tumor suppressor genes in an *in vivo* mouse cancer model, while Ren et al., used a CRISPR based HCV infection specific reporter system to identify the genes, *CLDN1*, *OCLN* and *CD81* as essential for both cell to cell transmission and cell free entry [95, 96]. For a comprehensive look at the current capabilities of genome wide screens please see the recent review from Shalem et al. [97].

### CRISPR/Cas9 as a potential therapeutic

One of the most exciting possibilities for the application of this technology is in the field of gene therapy for the treatment of diseases with a Mendelian genetic mutation as the root cause. Duchenne muscular dystrophy (DMD) is caused by mutations in the dystrophin gene, which consists of 79 exons, located on the X chromosome, making it a potential candidate for gene therapy [98]. Researchers from the Hotta group at Kyoto University, Japan, have shown that exon knock-in in DMD-patient-derived cells was the most effective way to correct mutations in the dystrophin gene, compared to exon skipping and frameshifting technologies. After correcting the various mutations, they were able to differentiate the corrected iPSCs into skeletal muscle cells that expressed the full-length dystrophin gene [99]. CRISPR/Cas9 was also effective in preventing the DMD phenotype in a mouse model [100]. In addition, the recent creation of a DMD disease model in the rhesus monkey using CRISPR/Cas9 could lead to new therapeutic approaches for this disease [90].

The CRISPR system, which has been adapted for numerous uses, is based on a protozoan defense system against viral infection, and therefore, it is a natural progression to co-opt this system as an anti-viral therapeutic. One example is the targeting of Hepatitis B virus. While current technologies are able to inhibit the covalently closed circular viral DNA template, they are not able to destroy it. However, Zhen et al. were able to target the surface antigen of HBV specifically to reduce the amount of antigen secreted in both cell culture and mouse serum, and almost completely eliminate its presence in mouse liver tissue [101]. In addition to work on HBV, CRISPR/Cas9 was also able to disrupt latent HIV infection in induced pluripotent stem cells and protect against new HIV infection as a proof of concept for the use of this technology as a treatment/defense application [102].

While the aforementioned studies have shown that CRISPR/Cas9 can be used to correct genetic maladies as a proof of concept, treatment would need to be delivered to developed individuals rather than at the embryonic stage, as is the case for the creation of transgenic mice. One of the first groups to show that genetic modifications could be made in adult animals was the Anderson lab at MIT. They were able to show that a hydrodynamic injection of CRISPR/Cas9 components could correct a mutation in the *Fah* gene in the hepatocytes of a transgenic mouse model of human tyrosinemia [103]. In addition to these results, the Chiarle lab at Harvard University used intratracheal and intrapulmonary delivery of CRISPR/Cas9 lentiviral vectors in adult male mice to cause a chromosomal rearrangement of the EML4 and Alk genes in order to mimic the inversion responsible for creating the desired EML4-ALK fusion protein. The engineered EML4-Akl gene fusion resulted in lung tumors within two months after the introduction of the recombinant virus [104]. These results indicate that genome editing in adult animals is indeed possible with CRISPR/Cas9.

### Additional uses of CRISPR/Cas9

The advent of CRISPR/cas9 has improved our ability to study more difficult areas of genetics, such as epigenetics. The use of dCas9 with epigenetic effector or repressor molecules is an area of research that was largely observational and can now move into a more experimental form of research with loss-of-function and gain-of-function studies now more possible than ever before [105]. Another interesting use of CRISPR/Cas9 is the investigation of a specific genomic region. Visualization of specific regions of DNA to better understand their spatiotemporal organization can be accomplished through fusing dCas9 to fluorescent protein such as EGFP [106]. This technique has recently been expanded to allow for multicolor applications [107]. Identification of the proteins bound to certain regions of the genome can be difficult but the engineered DNA-binding molecule-mediated chromatin immunoprecipitation method (enCHIP) developed by the Fuji lab was able to use the dCas9 fused to a protein tag to purify specific regions of the genome [108]. Utilizing mass spectrometry, they were able to identify the proteins associated with the immunoprecipitated regions of chromatin.

### Concluding remarks and future directions

Perhaps the ultimate goal of RNA guided genome editing is the development of therapies for both monogenic and polygenic diseases. The biggest roadblock in the development of these technologies is the issue of safety. It has been shown that the CRISPR/Cas9 technology can create the necessary changes to correct certain genetic defects in cells cultured from diseased individuals. However, it is not clear what the systemic effects of the treatment would be when administered to an individual. Therefore, the next step in the development of these treatments will be corresponding advances in methods to ensure that only the diseased cells of interest would be able to express the CRISPR/Cas components. Evidence of this possibility has been published recently in zebrafish [109]. Moreover, the issue of off-target effects still looms large as a source of considerable apprehension about the use of CRISPR/Cas9 for genome editing in humans. However, the development of nickases and advanced screening via next generation sequencing has helped decrease the likelihood of negative effects due to off-target effects. Another area where this system could be improved is in multiplex implementation. The Frew lab at the University of Zurich has attempted to simplify and optimize this approach through the development of their multiple lentiviral expression system (MuLE) that works as a modular vector system for easy editing of multiple areas at once [110].

The CRISPR/Cas9 system of genome editing and manipulation has proved to be an exciting new development for the fields of molecular biology and translational medicine. The ability to use RNA guided endonucleases to target virtually any area of an organism’s genome has lead to significant improvements in our ability to study various aspects of the genome, including the importance and function of the genes themselves, as well as the regulatory components that control them. This technology has shown the potential to bring about a new age of gene therapy that could lead to the treatment of diseases that were previously thought untreatable.

## Abbreviations

BAC: Bacterial artificial chromosome
Cas: CRISPR-associated proteins
Cas9: Native Cas9 nuclease
Cas9n: Cas9 nickase (containing only one functional catalytic domain)
CIBN: Calcium and integrin-binding protein 1 N terminal fragment
CRISPR: Clustered, regularly interspaced short palindromic repeats
CRISPRa: CRISPR activation
CRISPRi: CRISPR inhibition
CRISPRi/a: CRISPR inhibition and activation system
crRNA: CRISPR RNA
dCas9: “Dead” Cas9 (containing no functional catalytic domains)
DMD: Duchenne muscular dystrophy
DSB: Double stranded break
enCHIP: Engineered DNA-binding molecule mediated chromatin immunoprecipitation
fCas9: dCas9 fused to *Fok*I catalytic domain
gRNA: Guide RNA
HDR: Homology directed repair
iCRISPR: Inducible CRISPR
KRAB: Kruppel-associated box domain of kox1
LACE: Light-activated CRISPR-Cas9 effector system
LITES: Light inducible transcriptional effectors
MCR: Mutagenic chain reaction
MULE: Multiple lentiviral expression system
NHEJ: Non-homologous end joining
NmCas9: Cas9 from *Neisseria menigitidis*
PAM: Protospacer adjacent motif
SaCas9: Cas9 from *Staphylococcus aureus*
sgRNA: Single guide RNA
SpCas9: Cas9 from *Streptococcus pyogenes*
ssODN: Single-strand oligodeoxynucleotide
TALEN: Transcription activator-like effector nuclease
tracrRNA: Trans-activating RNA
ZFN: Zinc finger nuclease
